# 超高效液相色谱-串联质谱法快速测定动物源性食品中卢巴贝隆残留

**DOI:** 10.3724/SP.J.1123.2025.02006

**Published:** 2026-04-08

**Authors:** Cheng YANG, Weixia ZHU, Yafeng LIU, Hongwei ZHANG, Wei WEI, Kai HU

**Affiliations:** 1.郑州海关技术中心，河南 郑州 450003; 1. Zhengzhou Customs District，Zhengzhou 450003，China; 2.青岛海关技术中心，山东 青岛 266002; 2. Qingdao Customs District，Qingdao 266002，China; 3.河南中医药大学，河南 郑州 450046; 3. Henan University of Chinese Medicine，Zhengzhou 450046，China

**Keywords:** 卢巴贝隆, 固相萃取, 超高效液相色谱-串联质谱, 牛肉, lubabegron （LUB）, solid-phase extraction （SPE）, ultra performance liquid chromatography-tandem mass spectrometry （UPLC-MS/MS）, beef

## Abstract

建立了基于固相萃取-超高效液相色谱-串联质谱法（SPE-UPLC-MS/MS）快速准确测定动物源性食品中卢巴贝隆（LUB）残留的检测方法。样品经含0.5%甲酸的乙腈溶液提取后，采用PRiME HLB小柱进行固相萃取净化，采用0.1%甲酸水溶液-0.1%甲酸乙腈体系为流动相，在Shim-pack GIST C18-AQ 色谱柱（100 mm×2.1 mm， 2.7 μm）上进行梯度洗脱分离，流速0.3 mL/min，采用电喷雾正离子、多反应监测（MRM）模式进行检测，外标法定量。在最优的实验条件下，不同动物源性食品基质中LUB在各自的质量浓度范围内具有良好的线性关系，相关系数（*r*）均大于0.99，检出限（LOD）和定量限（LOQ）分别为0.4~1.0 μg/kg和1.0~2.0 μg/kg。在低、中、高3个加标水平下，LUB的加标回收率为81.5%~116.5%，相对标准偏差为2.0%~8.2%。该方法简单、快速，灵敏度高，可实现动物源性食品中LUB残留的快速检测，为进出口动物源性食品中LUB残留的日常检测提供了技术支撑。

## Abstract (en)

Lubaberone （LUB） is the first U S Food and Drug Administration （FDA） approved feed additives for reducing gaseous emissions from animals or their wastes， and the intake of animal-derived foods is an important source of human exposure to LUB. However， the lack of applicable analytical methods makes it difficult for regulatory authorities to monitor LUB in animal-derived foods. There is an urgent need to establish efficient and accurate methods for the analysis of LUB in animal-derived foods. In this study， a method was developed for the determination of LUB from six types of typical animal-derived foods （beef， bovine liver， bovine fat， mutton， sheep liver and sheep fat） by ultra performance liquid chromatography-tandem mass spectrometry （UPLC-MS/MS）. The effects of extraction solvent types， extraction methods， extraction time and purification cartridges on the recovery of LUB were investigated， and the optimal conditions for sample pretreatment were confirmed. The homogenized samples were extracted using 0.5% formic acid acetonitrile via ultrasonication for 10 min， and then filtered by high-speed centrifugation， and the extracted solution was cleaned using SPE with a PRiME HLB cartridge. The LUB was separated on a Shim-pack GIST C18-AQ chromatographic column （100 mm×2.1 mm， 2.7 μm） with 0.1% formic acid aqueous solution-0.1% formic acid acetonitrile as mobile phases at a flow rate of 0.3 mL/min， and detected in positive ion switching mode （ESI^+^） with multiple reaction monitoring （MRM） scanning， and quantitatively analyzed using the external standard method. Under the optimized experimental conditions， LUB in different animal-derived food matrices showed good linearity within their respective linear concentration ranges with the correlation coefficients （*r*） greater than 0.99. The limits of detection （LODs） and the limits of quantification （LOQs） were 0.4‒1.0 μg/kg and 1.0‒2.0 μg/kg， respectively. The recoveries of LUB spiked at low， medium and high levels ranged from 81.5% to 116.5% with relative standard deviations （RSDs） of 2.0%‒8.2%. The method is simple， rapid and highly sensitive， which can enable the analysis of LUB residues in animal-derived foods， and provides analytical technology support for the daily detection of LUB residues in imported and exported animal-derived foods.

卢巴贝隆（lubabegron，LUB，C_29_H_29_N_3_O_3_S，CAS号：391920-32-4），化学名为2-［4-［2-［［（2*S*）-2-羟基-3-（2-噻吩-2-基苯氧基）丙基］氨基］-2-甲基丙基］苯氧基］吡啶-3-甲腈，是一种*β*-肾上腺素受体调节剂，于2021年被美国食品和药物管理局（FDA）批准的首个用于减少动物或其排泄物气体排放的饲料添加剂^［1，2］^。其主要通过激活*β*_3_-肾上腺素受体，促进脂肪的分解和增加细胞能量消耗，改善动物的生长性能，减少脂肪沉积，降低氨气的产生，对畜牧业的环境可持续性产生了积极影响，同时LUB还对反刍动物的球虫病具有良好的治疗效果^［3］^。LUB残留可通过食物链进入人类体内，可能会对人类健康造成不良影响，随着LUB在畜牧业的应用，其残留引发的动物食品安全问题也逐渐受到关注。因此亟需建立针对动物源性食品中LUB残留的高效分析方法。

目前不同国家和地区对于LUB在动物源性食品中的残留限量制定了相关的限量标准。如日本将牛肉、牛脂肪、牛肝和牛肾等动物组织中的LUB残留限量设定为0.01~0.2 mg/kg^［4］^，但LUB的研究报道现主要集中于其工业合成和药理应用，针对LUB检测的分析方法报道还较少，Park等^［5］^构建了比目鱼、鳗鱼和虾肉中59种兽药残留的液相色谱-串联质谱分析方法，该方法主要利用水-乙腈（1∶4，体积比）混合溶液为提取溶剂对目标物进行提取，浓缩复溶后进行LC-MS/MS分析，其中LUB的定量限和检出限分别为1.0 µg/kg和0.3 µg/kg，此外尚未见相关报道。关于动物源性食品中兽药残留的检测方法主要有酶联免疫吸附测定法^［6］^、气相色谱-质谱法^［7］^、液相色谱法^［8］^和高效液相色谱-质谱法^［9］^等，其中高效液相色谱-串联质谱法灵敏度高，定性能力强，是目前分析兽药残留的首选方法。但由于动物源性食品中富含蛋白质、无机盐和脂肪等复杂基质，一般在分析时需要进行样品前处理。常见前处理方法包括固相萃取^［10，11］^、QuEChERS^［12，13］^、固相微萃取^［14］^和加速溶剂萃取^［15］^等。固相萃取方法具有操作简单、富集效果好、分离效率高、适用样品类型多和易于自动化等优点，能有效去除脂肪、蛋白质和有机酸等干扰基质，可实现高通量处理，适合大批量样品的分析检测。

本研究基于固相萃取技术结合超高效液相色谱-串联质谱法（UPLC-MS/MS），以动物源性食品中LUB为研究对象，通过优化前处理条件，建立了一种测定动物源性食品中LUB残留的分析方法，并对分析方法性能进行考察。该方法前处理简单，适用性强，可为动物源性食品中LUB的安全监测和控制提供技术支撑。

## 1 实验部分

### 1.1 仪器、试剂和材料

ACQUITY UPLC超高效液相色谱仪（美国Waters公司），三重四极杆线性离子阱质谱仪（美国AB SCIEX公司），KQ-300DE型数控超声波清洗器（昆山市超声仪器有限公司），WIGGENS Vortex 3000-Elite涡旋振荡器（北京维根技术有限公司），24位固相萃取装置（上海安谱实验科技股份有限公司），CF15RXII离心机（日本Hitachi公司），112N-EVAP112氮吹仪（美国Organo-mation Associates公司），0.22 μm有机滤膜（天津津腾公司），PRiME HLB固相萃取柱（150 mg/6 mL，美国Waters公司）。

卢巴贝隆（对照品，纯度≥99%，加拿大TRC公司），乙酸铵、乙腈和甲醇（色谱级，美国Fisher公司），实验用超纯水（18.2 MΩ·cm）由Milli-Q超纯水制备系统制备（美国Millipore公司），牛肉、牛肝、牛脂肪、羊肉、羊肝和羊脂肪样品购自当地市场，于‒20 ℃冰箱中储存。

### 1.2 溶液配制

标准储备液的制备：精密称取LUB对照品10.0 mg于100 mL容量瓶中用乙腈定容至刻度，配制成100.0 μg/mL的储备液，于‒20 ℃冰箱中避光保存。准确吸取适量标准储备液，用乙腈稀释，配制成质量浓度为1.0 μg/mL的混合标准中间液。

基质标准工作液：将空白基质样品经过前处理得到的溶液作为稀释溶剂，吸取适量1.0 μg/mL标准溶液，用空白样品提取液进行逐级稀释，配制成系列质量浓度的基质匹配混合标准溶液。

### 1.3 样品处理

将适量样品进行充分匀浆处理后，称取5.0 g于50 mL具塞塑料离心管中，加入10 mL 0.5%甲酸乙腈溶液和1 g NaCl，涡旋混匀30 s，超声提取10 min，8 000 r/min离心10 min后，取上清液加水定容至10 mL，待净化。取5 mL待净化液过PRiME HLB固相萃取小柱，控制流速1 mL/min，待柱子干涸后，用 3 mL 10%甲醇水溶液淋洗，弃去淋洗液，抽干，用3 mL 5%氨化甲醇进行洗脱，收集洗脱液，于40 ℃氮吹至近干，1 mL 0.1%甲酸水溶液-0.1%甲酸乙腈（50∶50， 体积比）复溶，过0.22 μm滤膜，供UPLC-MS/MS检测。

### 1.4 仪器条件

#### 1.4.1 色谱条件

色谱柱：Shim-pack GIST C18-AQ（100 mm×2.1 mm， 2.7 μm），柱温50 ℃。流动相为0.1%甲酸水溶液（A）和0.1%甲酸乙腈（B），流速：300 µL/min。梯度洗脱程序：0~2.0 min，2%B~40%B；2.0~6.0 min，40%B；6.0~6.10 min，40%B~95%B；6.10~9.0 min，95%B；9.0~9.10 min，95%B~2%B；9.10~12.0 min，2%B。进样量：10 μL。

#### 1.4.2 质谱条件

电喷雾离子源正离子电离模式（ESI^+^），毛细管电压：5 500 V；入口电压：10 V；雾化气压力：344.75 kPa；气帘气压力：172.38 kPa；加热辅助气压力：379.23 kPa；离子源温度：550 ℃。LUB的监测离子对及质谱参数如表1所示。

**表1:** LUB的保留时间和质谱参数

Compound	Retention time/min	Parent ion （*m/z*）	Product ion （*m/z*）	DP/V	CE/eV
LUB	7.90	500.0	250.3^*^	85	28
			215.2	85	40

* Quantitative ion； DP： declustering potential； CE： collision energy.

## 2 结果与讨论

### 2.1 质谱条件优化

将100 ng/mL的LUB直接注射到质谱仪中进行质谱条件优化。在正离子模式下对LUB进行扫描，观察到LUB的前体离子与质子化的［M+H］^+^加合物一致，对应的［M+H］^+^准分子离子峰为*m/z* 500.0。通过子离子扫描，LUB的主要碎片离子*m/z*为250.3、215.2、209.0、177.2和196.2等，其中*m/z* 250.3的子离子包含噻吩环和部分苯基取代的结构单元，其响应强度最高，该部分结构在裂解过程中相对稳定，因此用作定量离子对，其次*m/z* 215.2用作定性离子对。质谱条件中的去簇电压和碰撞能量对离子丰度具有较大影响，对方法灵敏度有决定性作用。当碰撞能量为28 eV时，LUB离子对*m/z* 500.0/250.3强度最高，当碰撞能量为40 eV时，LUB的离子对*m/z* 500.0/215.2强度最高。考察了5~180 V范围内不同去簇电压对LUB响应度的影响。结果表明，当去簇电压为85 V时，LUB子离子响应强度最高。LUB可能的质谱裂解途径见图1。

**图1:**
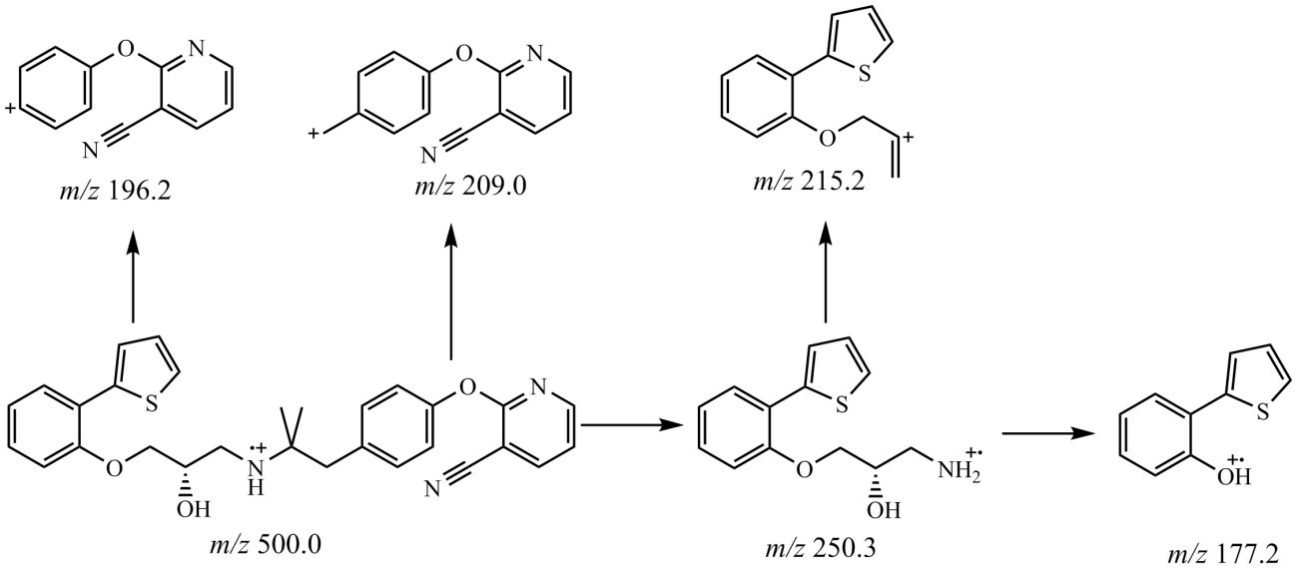
LUB可能的质谱裂解途径

### 2.2 色谱条件优化

实验比较了Poroshell 120 EC-C18（100 mm×3.0 mm，2.7 μm）和Shim-pack GIST C18-AQ（100 mm×2.1 mm， 2.7 μm）色谱柱的分离性能，结果显示，选用Shim-pack GIST C18-AQ色谱柱进行分离时，LUB的响应更高且保留时间更长，有利于目标物与基质成分的分离，因此选用Shim-pack GIST C18-AQ色谱柱进行LUB的分离。

进一步比较了水-乙腈、0.1%甲酸水溶液-乙腈、0.1%甲酸水溶液-0.1%甲酸乙腈3种不同流动相体系对LUB分离效果的影响。实验结果显示，当采用0.1%甲酸水溶液-0.1%甲酸乙腈作为流动相时，LUB展现出了较好的分离效果和较高的峰高，图2为优化条件下加标牛肉样品中LUB的MRM色谱图。

**图2:**
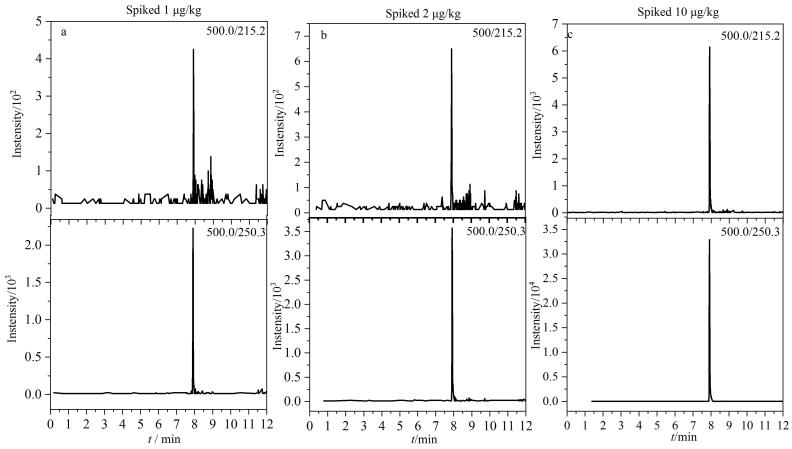
加标牛肉样品中LUB的MRM色谱图

### 2.3 前处理条件的优化

#### 2.3.1 提取条件的优化

动物源性食品富含脂肪和蛋白质等干扰物，选择合适的提取溶剂是提取LUB和避免共萃取基质干扰的关键之一。酸性溶剂可以使蛋白质沉淀，在酸性环境中，LUB的电离受到抑制，使其呈现分子状态，容易进入有机溶剂中被提取。前处理优化过程选取牛肉为样品基质，LUB的添加水平为100 μg/kg，实验设置3个平行样品，分别考察了含不同体积分数（0、0.1%、0.25%、0.5%、1.0%）甲酸的乙腈溶液作为提取溶剂对LUB回收率的影响，结果如图3a所示，添加甲酸后，LUB的回收率明显改善，随着甲酸体积分数的增加，LUB的回收率逐渐升高，在甲酸体积分数为0.5%时，LUB的回收率最高（100.60%）。因此，选取0.5%甲酸乙腈作为提取溶剂。

**图3:**
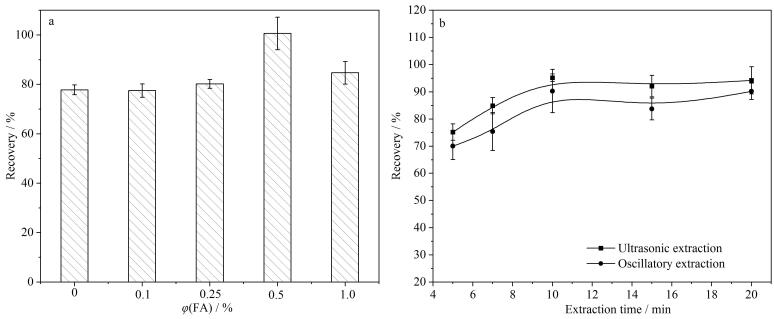
（a）乙腈中不同体积分数的甲酸和（b）提取方式、提取时间对LUB回收率的影响（*n*=3）

实验比较了超声和振荡两种提取方式对LUB回收率的影响，由图3b所示，在相同条件下，超声提取效率较好。此外，为考察提取时间对LUB回收率的影响，在相同条件下选取提取时间为5、7、10、15、20 min，结果显示，在超声提取10 min后，回收率均在90%以上，因此选择超声提取时间为10 min。

#### 2.3.2 净化条件的优化

从有机物分配系数（log *P*）来看，log *P*值越大，则疏水性越强，即极性越小。由ChemSpider数据库检索可知LUB的log *P*为4.58，极性相对较弱，属于亲脂性化合物。基于LUB的理化性质，本实验采取固相萃取方式对样品进行净化，实验中考察了Oasis WCX（150 mg/6 mL）、Oasis PRiME HLB（150 mg/6 mL）和Bond Elut C18（150 mg/6 mL）小柱的净化效果。选取牛肉为样品基质，LUB的添加水平为100 μg/kg，实验设置3个平行样品。结果显示，采用WCX和C18小柱进行样品净化时，LUB的回收率较差，其回收率均<60.0%（RSD≤10.3%），采用HLB小柱时LUB的回收率较高，为108.5%，RSD为7.6%，而采用更多填料的HLB（200 mg/6 mL）小柱，其回收率并无明显变化。此外，动物基质样品中含有蛋白质、脂肪、无机盐和磷脂类非极性干扰物，而Oasis PRiME HLB是一种新型的亲水亲脂平衡反相吸附剂，适用于酸性、碱性和中性化合物的高效吸附，且能够去除几乎所有的蛋白质、盐和磷脂类干扰物^［16‒19］^，在进行前处理分析时可直接上样，大大缩短分析时间，更适于大批量样品的处理。因此，选择PRiME HLB小柱对提取液进行净化。

### 2.4 基质效应

采用标准曲线法评估不同基质中的基质效应（ME），ME=（基质匹配标准曲线斜率/溶剂标准曲线斜率‒1）×100%。结果表明，LUB在牛脂肪和羊脂肪基质中基质效应属于中等或较高强度（42.0%~52.0%），在牛肉和羊肉基质中的影响可忽略不计（9.0%~12.0%），在牛肝和羊肝基质中基质效应属于中等强度（23.0%~32.0%） （表2）。因此，为提高定量结果的准确性，使用基质匹配标准曲线对LUB进行定量，以消除基质效应。

**表2:** LUB的线性范围、线性方程、相关系数、检出限、定量限和基质效应

Matrix	Linear range/（ng/mL）	Linear equation	*r*	LOD/（μg/kg）	LOQ/（μg/kg）	ME/%
Beef	1‒100	*Y*=5.74×10^4^ *X*‒5.00×10^4^	0.9998	0.5	1.0	12.2
Bovine liver	1‒100	*Y*=7.31×10^4^ *X*+1.90×10^4^	0.9980	0.5	1.0	32.1
Bovine fat	2‒100	*Y*=7.43×10^4^ *X*+1.52×10^4^	0.9930	1.0	2.0	42.0
Mutton	1‒100	*Y*=5.34×10^4^ *X*+6.07×10^4^	0.9987	0.5	1.0	9.0
Sheep liver	1‒100	*Y*=6.93×10^4^ *X*+2.35×10^4^	0.9963	0.4	1.0	23.2
Sheep fat	1‒100	*Y*=4.12×10^4^ *X*+3.38×10^4^	0.9972	0.7	1.0	52.1

*Y*： peak area； *X*： mass concentration， ng/mL.

### 2.5 方法学考察

#### 2.5.1 线性范围、检出限和定量限

用空白基质提取液配制系列浓度的标准溶液，以LUB定量离子对的峰面积（*Y*）为纵坐标，质量浓度（*X*， ng/mL）为横坐标绘制标准曲线，分别以信噪比（*S/N*）≥3和信噪比（*S/N*）≥10所对应的质量浓度作为LUB的检出限（LOD）和定量限（LOQ）。如表2所示，在6种动物源性食品基质中，LUB具有良好的线性关系，相关系数（*r*）均大于0.99，检出限和定量限分别为0.4~1.0 μg/kg和1.0~2.0 μg/kg。

#### 2.5.2 加标回收率和精密度

选择不含待测物的空白样品进行加标回收试验，分别添加低、中、高3个加标水平，每个加标水平平行测定6次，考察该方法的准确度和精密度。如表3所示，LUB的平均回收率为81.5%~116.5%，相对标准偏差（RSD）为2.0%~8.2%，表明方法的准确度和精密度良好，满足分析要求，可用于LUB残留的准确定量。

**表3:** 不同基质中LUB的加标回收率和RSD（*n*=6）

Matrix	Spiked/（μg/kg）	Found/（μg/kg）	Recovery/%	RSD/%
Beef	1	1.1	110.0	8.1
	2	2.0	101.0	3.9
	10	9.4	94.1	2.1
Bovine liver	1	0.8	84.3	3.0
	2	1.6	81.5	2.9
	10	11.2	112.0	2.4
Bovine fat	2	1.9	94.8	2.6
	4	3.8	95.2	8.2
	20	20.5	102.5	2.0
Mutton	1	0.9	95.4	5.9
	2	1.9	95.7	6.5
	10	9.8	98.5	4.8
Sheep liver	1	1.1	113.4	3.9
	2	1.7	89.2	6.0
	10	8.4	84.5	5.1
Sheep fat	1	0.8	87.4	8.2
	2	2.1	116.5	5.0
	10	9.6	96.4	4.6

### 2.6 实际样品的检测

为评价该方法的有效性，采用所建立的分析方法对10批不同来源的牛肉、牛肝、牛脂肪和羊肉等样品进行分析检测，结果均未检测出LUB残留。

## 3 结论

本研究基于酸化溶剂提取、固相萃取净化及超高效液相色谱-串联质谱法建立了一种测定动物源性食品中LUB残留的分析方法。该方法快速高效，准确度好，灵敏度高，重复性好，可满足动物源性食品日常批量检测的需求，为动物源性食品中LUB的检测和研究提供新思路和新方法，并为市场监管部门的风险监测和日常防控提供了技术支持。尽管如此，该方法仍具有一些局限性。一方面，由于LUB缺乏相应的内标物，分析结果的准确性可能受到仪器响应波动、样品基质效应和进样误差等多种因素的影响；另一方面，该方法尚未实现自动化，无法实现高通量分析，需在今后的工作中进一步改进。

## References

[1] US Food and Drug Administration （FDA） . FDA Approves First Animal Drug that Reduces Gas Emissions from an Animal or Its Waste. （2019-02-21） ［2025-02-11］. https://www.fda.gov/news-events/fda-brief/fda-brief-fda-approves-first-animal-drug-reduces-gas-emissions-animal-or-its-wastehttps://www.fda.gov/news-events/fda-brief/fda-brief-fda-approves-first-animal-drug-reduces-gas-emissions-animal-or-its-waste

[2] Hwang J H， Kube J C， Smith S B . J Anim Sci， 2022， 100（3）： 1 10.1093/jas/skac052 PMC9030222 35262701

[3] Kube J C， Holland B P， Word A B， et al . Transl Anim Sci， 2021， 5（3）： 1 10.1093/tas/txab137 PMC8439260 34532643

[4] World Trade Organization . The Proposed Maximum Residue Limits （MRLs） for Lubabegron Notified in G/SPS/N/JPN/1032. （2022-12-19） ［2025-02-11］. https://members.wto.org/crnattachments/2022/SPS/JPN/22_8670_00_e.pdfhttps://members.wto.org/crnattachments/2022/SPS/JPN/22_8670_00_e.pdf

[5] Park S， Park H， Kim J Y， et al . Food Anal Methods， 2024， 17： 61

[6] Li R， Lin Z J， Yang J Y， et al . Chinese Journal of Analytical Chemistry， 2018， 46（8）： 1321

[7] Li J， Ju X， Wang Y L， et al . Chinese Journal of Chromatography， 2023， 41（7）： 610 37387282 10.3724/SP.J.1123.2022.10010 PMC10311622

[8] Xue W， Li N， Zhang Z， et al . Talanta， 2022， 239： 123065 34875523 10.1016/j.talanta.2021.123065

[9] Sun Q， Liu J， Gou Y， et al . J Chromatogr A， 2025， 1744： 465726 39893914 10.1016/j.chroma.2025.465726

[10] Tang Y， Wen S， Cao W C， et al . Chinese Journal of Chromatography， 2025， 43（4）： 309 40133196 10.3724/SP.J.1123.2024.07010 PMC11966372

[11] Du X， Sun M Y， Weng J J， et al . Chemical Reagents， 2023， 45（10）： 110

[12] Tong X Z， Chen D Y， Feng J L， et al . Chinese Journal of Chromatography， 2023， 41（6）： 490 37259873 10.3724/SP.J.1123.2022.09020 PMC10245214

[13] Li Z M， Xie Y L， Ma C G， et al . Chemical Reagents， 2024， 46（6）： 99

[14] Zhan X W， Li P H， Xu L . Chinese Journal of Analytical Chemistry， 2023， 51（6）： 1033

[15] Wang Q X， Feng Q Y， Zhu X Q . Chinese Journal of Chromatography， 2023， 41（7）： 582 37387279 10.3724/SP.J.1123.2022.12015 PMC10311623

[16] Wang J Q， Ma L L， Cao Y H， et al . Chinese Journal of Analysis Laboratory， 2018， 37（3）： 306

[17] Zhang D， Liu B L， Zhao Z W， et al . Chinese Journal of Analytical Chemistry， 2023， 51（8）： 1343

[18] Qiu Q L， Chen X H， Pan S D， et al . Chinese Journal of Chromatography， 2022， 40（7）： 669 35791606 10.3724/SP.J.1123.2022.01017 PMC9404095

[19] Wang M Y， Chen Y C， Tu F Q， et al . Chinese Journal of Chromatography， 2020， 38（12）： 1423 34213257 10.3724/SP.J.1123.2020.06017

